# Factors leading to open revision surgery after trans-sacral canal plasty for lumbar spine disease

**DOI:** 10.3389/fsurg.2024.1370754

**Published:** 2024-05-30

**Authors:** Daigo Arimura, Akira Shinohara, Shunsuke Katsumi, Shintaro Obata, Taku Ikegami, Naomu Sawada, Keiichiro Mori, Mitsuru Saito

**Affiliations:** ^1^ Department of Orthopedic Surgery, The Jikei University School of Medicine, Tokyo, Japan; ^2^ Department of Urology, The Jikei University School of Medicine, Tokyo, Japan

**Keywords:** trans-sacral canal plasty (TSCP), Racz catheter, adhesiolysis, minimally invasive spine surgery (MISS), lumbar spine disease

## Abstract

Trans-sacral canal plasty (TSCP) is a minimally invasive lumbar spine surgery under local anaesthesia. TSCP is expected to be effective regardless of whether the patient has had previous surgery. However, there are cases in which open revision surgery is required after TSCP. This study aimed to identify risk factors for open revision surgery after TSCP in order to determine surgical indications and limitations. A retrospective case-control study was conducted in patients who underwent TSCP for lumbar spine disease. Data of 112 patients were analysed. During an observation period of 7–23 months, 34 patients (30.4%) required open revision surgery and 78 (69.6%) did not. The following patient background characteristics were investigated: age, sex, body mass index (BMI), diagnosis, history of spine surgery and the institution where the surgery was performed. Comorbidities were scored using the Elixhauser Comorbidity Index. Preoperative imaging parameters were investigated, including the lesion level (L4/5, L5/S1, other), presence of intervertebral instability, dural sac area, presence of bony stenosis and presence of epidural lipoma. Multivariate analysis revealed that intervertebral instability (odds ratio 2.56, confidence interval 1.00–6.51, *p* = 0.046) and a narrow dural sac area (odds ratio 0.98, confidence interval 0.97–0.99, *p* = 0.002) were significant risk factors for open revision surgery after TSCP.

## Introduction

In recent years, minimally invasive spine surgery has been increasingly used and contributed much to the treatment of spinal disorders (1–4). Minimally invasive spine surgery has many advantages, including smaller skin incisions, less blood loss and more rapid patient recovery (4), and has the potential to avoid conventional surgery and be cost-effective (5, 6).

One type of minimally invasive spine surgery is epidural lysis of adhesions, which is performed for back pain and leg pain (7) caused by failed back surgery syndrome (8–11) and lumbar spinal canal stenosis (10, 12) and when treatments such as epidural steroid injections are ineffective. The demand for this treatment is increasing as the population ages, but there has been an increase in the number of patients with complications. Epidural debridement has also been reported to be cost-effective (10).

Trans-sacral canal plasty (TSCP) is one of the methods that can be used for epidural lysis of adhesions and was recently described by the Intraspinal Canal Treatment (ISCT) Study Group, which is a subcommittee of the Minimally Invasive Spinal Treatment Society (8, 13). TSCP can be performed under local anaesthesia using a trans-sacral approach with a 2.65-mm disposable steerable catheter. A distinctive feature of this treatment is that mechanical adhesiolysis can be achieved by moving the highly manoeuvrable catheter from side to side. TSCP has been reported to be effective whether or not there is a history of previous spine surgery (8). At our hospital, TSCP has become the first-line treatment for patients with lumbar spine conditions that do not respond to conservative treatment, such as lumbar spinal stenosis and lumbar disc herniation (13). However, more than a few patients experience pain flares after TSCP and require further spine surgery. Therefore, there is a need to be able to identify candidates for TSCP based on preoperative background and imaging characteristics.

The purpose of this study was to investigate the surgical indications and limitations of TSCP and to identify the factors leading to open revision surgery (14).

## Methods

This retrospective review of patients who underwent TSCP between October 2020 and February 2022 was conducted at the Jikei University Hospital and its affiliated hospital. The study was carried out in accordance with principles laid down in the Declaration of Helsinki, and the study protocol was approved by the Jikei University School of Medicine Ethics Committee [30–115 (9136)]. All patients provided written informed consent.

TSCP was performed for the following lumbar spine conditions causing low back or leg pain: lumbar spinal stenosis, lumbar degenerative spondylolisthesis, lumbar spondylolysis, lumbar disc herniation, lumbar disc disease, and sacral cysts. Fractures, tumours, and adult spinal deformities were excluded. Patients with emergent symptoms such as paralysis or bladder and rectal disorders were also excluded from the TSCP indications. Also, TSCP was proposed for patients who did not respond to conservative treatment over a certain period of time and who would have conventionally been recommended open surgery. Therefore, lumbar disc disease and sacral cysts were excluded from this series because these are not indications for open surgery, even if they are not improved by TSCP. Patients were excluded if they had insufficient data as a result of death or loss to follow-up.

TSCP was performed using the following techniques. The patient was placed in the prone position, local anaesthesia was administered around the sacral hiatus, a small incision was made with a scalpel, and an introducer was placed into the sacral hiatus under fluoroscopic guidance. After placement in the epidural space, the catheter was advanced upwards along the ventral or dorsal aspect of the epidural canal to the lesion. Under fluoroscopic guidance, mechanical adhesiolysis was performed by moving the catheter left to right and up and down. Contrast medium was injected to detach adhesions from the dural canal and around the nerve roots and to confirm detachment of the adhesions. A mixture of saline (6 ml), 1% mepivacaine hydrochloride (4 ml), and dexamethasone (3.3 mg) was injected at the end of the procedure.

The following patient background characteristics were investigated: age, sex, BMI, diagnosis, history of spine surgery and the institution where the surgery was performed. Comorbidities were scored using the Elixhauser Comorbidity Index (ECI) (15–17). Preoperative imaging parameters were investigated, including the lesion level (L4/5, L5/S1, other), presence of intervertebral instability [change of more than 4 mm anteriorly or 2 mm posteriorly on dynamic radiographs (14)], dural sac area (mm^2^, calculated using ImageJ software [National Institutes of Health, Bethesda, MD] (13)), presence of bony stenosis (bony prominence protruding more than 5 mm towards the dural sac) and presence of epidural lipoma (ventral side of the dural canal, determined to be the cause of the stenosis). For the dural sac area, we selected the higher-intensity area that we considered the most significant cause of symptoms in each patient. We also investigated a visual analogue scale (VAS, 0–100 mm) for low back pain and leg pain recorded preoperatively, immediately after surgery and 1, 3, 6 and 12 months postoperatively.

The patients were divided into those who had not undergone open revision surgery [RS(−) group] and those who had undergone open revision surgery [RS(+) group] by the final follow-up (September 30, 2022). Background characteristics and imaging parameters were then compared between the two groups.

All statistical analyses were performed using GraphPad Prism (version 5; GraphPad Software Inc., La Jolla, CA) and Stata (version 14; StataCorp LLC, College Station, TX). All tests were two-sided and a *p-*value of <0.05 was considered statistically significant. Patient demographics were compared between the two groups using the independent *t*-test and chi-squared test. Univariable and multivariable logistic regression analyses were performed to investigate the association of pretreatment factors with the need for further surgery. The optimal cutoff value was defined by creating a receiver-operating characteristic (ROC) curve to yield the highest Youden index value. Briefly, the Youden index provides the optimal cutoff for a continuous variable by showing the score that offers the best trade-off between sensitivity and specificity. The area under the ROC curve was calculated to determine the discrimination ability of the logistic regression models. The VAS scores were compared between groups by repeated-measures analysis of variance followed by *post hoc* analysis with Bonferroni adjustment using IBM SPSS Statistics (version 25; IBM Corp., Armonk, NY).

## Results

### Demographics

**Figure 1:**
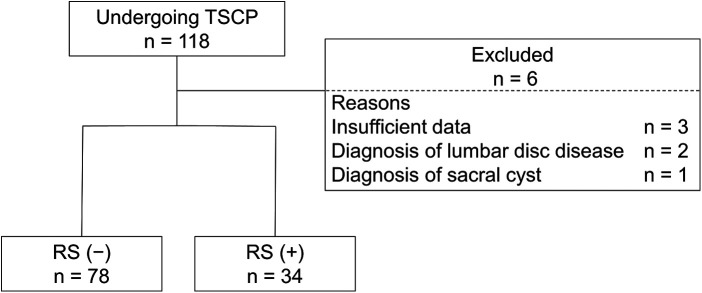
Flow diagram showing the patient selection process. TSCP, trans-sacral canal plasty; RS, open revision surgery.

A total of 118 patients underwent TSCP during the study period. After exclusion of 6 patients with incomplete data (*n* = 3), a diagnosis of lumbar disc disease (*n* = 2) or a diagnosis of sacral cyst (*n* = 1), the remaining 112 patients were divided into those who did not undergo open revision surgery [RS(−) group, *n* = 78, 69.6%] and those who did [RS(+) group, *n* = 34, 30.4%] (Figure 1). The mean follow-up duration ± standard deviation (SD) was 15.2 ± 4.6 months. The mean duration ± SD between TSCP and open revision surgery was 28 ± 40 days.

Intraoperative complications were headache and discomfort in 1 case and catheter breakage in another, giving a rate of 1.7% (2/112). Postoperative complications included stress-related gastrointestinal problems in 2 cases and transient neuropathy in 3 cases (5/112, 4.4%). The 3 cases of transient neuropathy had temporary worsening of leg pain and numbness after TSCP postoperatively, but all patients had spontaneous resolution within 1 month, and this complication was possibly due to irritation from catheter manipulation during TSCP.

There were significant between-group differences in the institution where the procedure was performed (*p* = 0.03), intervertebral instability status (*p* = 0.02) and dural sac area (*p* = 0.001) (Table 1). However, there was no significant in age, sex, body mass index (BMI), diagnosis, history of spine surgery, ECI, lesion level, bony stenosis status or epidural fat status between the groups.

**Table 1:** Patient characteristics.

Variables	RS(−) (*n* = 78)	RS(+) (*n* = 34)	Entire cohort (*N* = 112)	*p*-value
Age, years (mean ± SD)	72 ± 13.9	66 ± 11.9	70 ± 13.5	0.08
Sex				0.68
Male, *n* (%)	43 (55.1)	17 (50.0)	60	
Female, *n* (%)	35 (44.9)	17 (50.0)	52	
BMI (mean ± SD)	23.3 ± 3.9	24.6 ± 3.7		0.26
Diagnosis				
Lumbar spinal canal stenosis	57 (73.1)	24 (70.6)	81	0.32
Lumbar degenerative spondylolisthesis	10 (12.8)	3 (8.8)	13	
Lumbar spondylolysis	3 (3.8)	0 (0.0)	3	
Lumbar disc herniation	8 (10.3)	7 (20.6)	15	
History of spine surgery				0.06
No	55 (70.5)	30 (88.2)	85	
Yes	23 (29.5)	4 (11.8)	27	
Institution				0.03*
University hospital	31 (39.7)	6 (17.6)	37	
Affiliated hospital	47 (60.3)	28 (82.4)	75	
ECI (mean ± SD)	0.824 ± 0.04	0.824 ± 0.03		0.13
Intervertebral instability				0.02*
No	54 (69.2)	15 (44.1)	69	
Yes	24 (30.8)	19 (55.9)	43	
Dural sac area (mean ± SD)	100.4 ± 52.9	72.8 ± 27.9		0.001*
Level of lesion				0.9
L4/5	57 (73.1)	24 (70.6)	81	
L5/S1	16 (20.5)	7 (20.6)	23	
Other (L2/3 or L3/4)	5 (6.4)	3 (8.8)	8	
Bony stenosis				0.56
No	68 (87.2)	28 (82.4)	96	
Yes	10 (12.8)	6 (17.6)	16	
Epidural fat				0.43
No	71 (91.0)	33 (97.1)	104	
Yes	7 (9.0)	1 (2.9)	8	

BMI, body mass index; ECI, elixhauser comorbidity index; SD, standard deviation.

* Statistical significance (*p* < 0.05).

### Comparison of risk of open revision surgery between the groups

Univariate analysis of various patient and imaging parameters was performed to identify risk factors leading to open revision surgery after TSCP. The results showed that institution where the procedure was performed (odds ratio [OR] 3.08, confidence interval [CI] 1.14–8.30, *p* = 0.03), intervertebral instability (OR 2.85, CI 1.24–6.54, *p* = 0.01) and a narrow dural sac area (OR 0.98, CI 0.97–0.99, *p* = 0.0005) were significant influencing factors (Table 2). In multivariate analysis, intervertebral instability (OR 2.56, CI 1.00–6.51, *p* = 0.046) and a narrow dural sac area (OR 0.98, CI 0.97–0.99, *p* = 0.002) were factors that significantly affected the risk of open revision surgery (Table 2). The cutoff value calculated from the ROC curve for dural sac area was 93.9 mm^2^.

**Table 2:** Univariate and multivariate analysis of factors associated with open revision surgery after trans-sacral canal plasty.

	Univariate			Multivariate		
	OR	95% CI	*p*-value	OR	95% CI	*p*-value
Age (mean ± SD)	0.97	0.95–1.00	0.08	0.97	0.93–1.00	0.07
Sex	1.23	0.55–2.75	0.62			
Body mass index	1.06	0.96–1.18	0.26			
Diagnosis	1.18	0.82–1.71	0.89			
History of spine surgery	0.32	0.10–1.01	0.052	0.73	0.20–2.66	0.63
Institution	3.08	1.14–8.30	0.03*	2.59	0.84–8.02	0.09
ECI	65806.59	0.02–1.81e + 11	0.14			
Intervertebral instability	2.85	1.24–6.54	0.01*	2.56	1.00–6.51	0.046*
Dural sac area	0.98	0.97–0.99	0.0005*	0.98	0.97–0.99	0.002*
Level of lesion	1.14	0.60–2.18	0.7			
Bony stenosis	1.46	0.48–4.39	0.5			
Epidural fat	0.31	0.04–2.60	0.28			

CI, confidence interval; ECI, elixhauser comorbidity index; SD, standard deviation.

* Statistical significance (*p* < 0.05).

### VAS scores in the Rs(−) group

**Figure 2:**
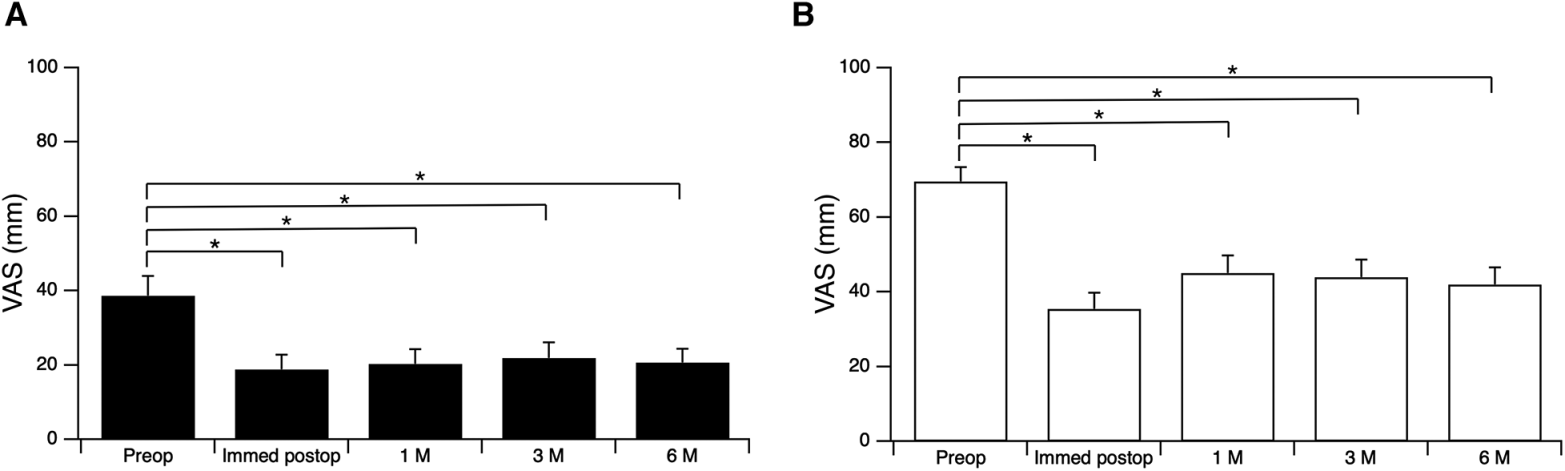
Visual analogue scale (VAS) scores preoperatively and immediately, 1, 3 and 6 months postoperatively. (**A**) Lumbar pain; (**B**) leg pain. *p < 0.001, repeated measures analysis of variance followed by *post hoc* Bonferroni adjustment. M, months.

In the RS(−) group, the mean VAS score (± standard error of the mean) for back pain and leg pain obtained preoperatively were compared with those obtained 6 months postoperatively for the 49 patients for whom data were available. The mean VAS score for lumbar pain decreased significantly from 38.7 ± 5.3 mm preoperatively to 18.9 ± 4.0 mm, 20.3 ± 3.9 mm, 21.9 ± 4.2 mm, and 20.7 ± 3.6 mm immediately and 1, 3 and 6 months postoperatively (Figure 2A). The VAS score for leg pain decreased significantly from 69.6 ± 4.0 mm preoperatively to 35.4 ± 4.3 mm, 45.1 ± 4.7 mm, 44.0 ± 4.7 mm and 41.9 ± 4.5 mm immediately and 1, 3 and 6 months after surgery (Figure 2B). VAS scores were measured only for evaluation of the effect of TSCP so were not recorded after surgery in the RS(+) group.

## Discussion

Epidural adhesiolysis is an interventional treatment for lumbar spinal canal stenosis, lumbar disc herniation and postoperative lumbar spine syndromes (18, 19). Since 1989 when Racz et al. first performed this procedure using hypertonic saline (20, 21), it has been performed worldwide. The main mechanisms involved in exfoliation of epidural adhesions are removal of perineural adhesions that may aggravate neuralgia (22) and the washout effect of inflammatory cytokines present in the lesion after steroid injection (23).

A meta-analysis of two randomized controlled trials and four observational studies provided Level II evidence for the effectiveness of percutaneous adhesiolysis for lumbar spinal canal stenosis (10). In addition, according to a systematic review of nine RCTs, percutaneous adhesiolysis was effective for 1 year in the management of low back and leg pain in patients with lumbar spinal stenosis, lumbar disc herniation and postoperative pain syndrome (24). Another study demonstrated the effectiveness of epidural nerve adhesiolysis for low back and leg pain regardless of type of lumbar disc herniation (25). Other studies (26, 27) have focused on comparing physical therapy and epidural anaesthesia based on the assumption that epidural adhesiolysis has only an interventional role, but the data support the definite presence of adhesions even in preoperative patients with lumbar spinal stenosis or lumbar disc herniation. In addition, Yokosuka et al. performed epidural contrast imaging and evaluated CT in patients who had undergone adhesion release for severe lower back pain (28). The results showed morphological features of the epidural space that were not depicted on MRI. In addition, although the CT images were obtained after adhesiolysis, they revealed the presence of epidural connective tissue or adhesions (28). Thus, it is clear that adhesions were present in the epidural space, even in preoperative patients. During the TSCP procedure, we position the catheter ventral to the dural canal and flow contrast before dissection of the adhesions to ensure that the contrast is interrupted at the stenosis. Then, after sufficient dissection of the stenosis, we again flow contrast to confirm that it can pass through the stenosis.

Unlike conventional catheters such as the Racz, the catheter used for TSCP (myeloCath®, Biomedica Healthcare Ltd., Tokyo, Japan) (8) is movable with excellent operability and followability, and mechanical adhesiolysis is possible by simply moving it from side to side (13). Data from collaborative studies at other institutions have shown that TSCP improves back and leg pain regardless of whether the patient has had previous spine surgery and that it may be a treatment option for elderly and immunocompromised patients for whom multiple surgeries are not recommended (8). Therefore, in this study, in order to determine whether TSCP can be considered a definitive surgery or simply an interventional treatment, we aimed to clarify whether TSCP can avoid open revision surgery and identify the factors that may lead to additional surgery.

In this study, we evaluated the efficacy of TSCP by focusing on whether the patient underwent open revision surgery after TSCP. As in previous reports (29, 30), we considered including duration of symptoms and severity of preoperative leg and back pain as prognostic factors but were unable to do so owing to lack of sufficient data. We consider this to be a limitation of this study. In addition, dural sac area rather than spinal canal area was selected as a factor in the assessment of stenosis. Consistent with this, there are previous reports indicating that dural sac area is a more sensitive measurement parameter than spinal canal area (31) and that a narrow dural sac area on magnetic resonance images is associated with leg pain at 1 year after lumbar spine surgery (32). Further, in view of a report suggesting that stenosis caused by epidural lipomatosis can be relieved by dissection of adhesions (13), the presence or absence of epidural lipomatosis was also selected as a factor. Other limitations of this study are that not all factors were included in the multivariate analysis and collinearity between factors could not be considered.

Comparison of our RS(−) and RS(+) groups showed significant differences in terms of the facility where the patient underwent surgery (*p* = 0.03), intervertebral instability status (*p* = 0.03) and dural sac area (*p* = 0.03), but not in the other parameters. The finding on where the surgery was performed could reflect the fact that additional surgery can be organized in a timelier manner by affiliated hospitals than by university hospitals after the decision is made. Another possibility was that patients at the university hospitals and affiliated hospitals were more likely to have more complications, making it more difficult to select additional surgeries. However, there were no obvious differences in ECI between institutions. We considered adjusting for the variation between groups using the propensity score matching method but did not do so because it would have reduced the number of subjects.

**Figure 3:**
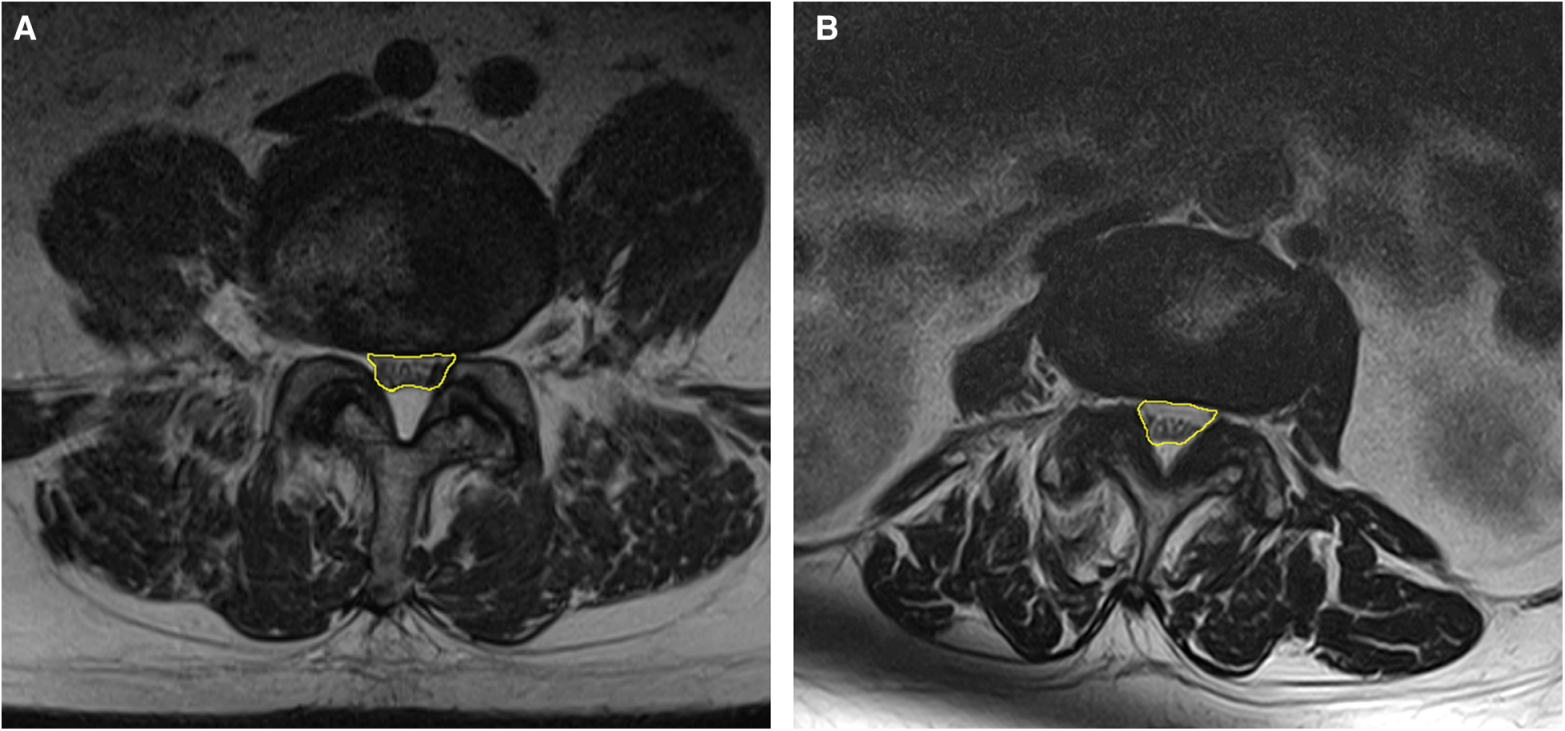
Representative examples of areas that are close to the cutoff value of 93.9 cm^2^. The dural sac area is indicated in yellow. (**A**) 93.7 mm^2^ at L4/5 (**B**) 93.9 mm^2^ at L3/4.

Potentially relevant patient background and imaging characteristics were examined in univariate analysis, and factors with *p*-values <0.1, namely, age, history of surgery, facility where surgery was performed, intervertebral instability and dural sac area, were selected for multivariate analysis. The results showed that presence of intervertebral instability (*p* = 0.046) and a narrow dural sac area (*p* = 0.002) were significant independent factors associated with the need for additional surgery, which is in contrast with the findings of a study of the effectiveness of the Racz catheter that identified a history of lumbar spine surgery and lumbar disc herniation to be prognostic factors (30). In the present study, a history of surgery and the diagnosed disease were not significant factors. The reason why a history of surgery was not a poor prognostic factor for additional surgery may reflect the fact that the catheter used for TSCP is easy to operate and has a good ability to dissect adhesions. However, the degree of stenosis, which was not significant in previous reports, was extracted as a significant factor in this study; one possible reason for this finding is that the degree of stenosis was evaluated quantitatively using the dural sac area instead of scoring into three levels (30). According to the ROC curve, the optimal cutoff value for the spinal sac area was 93.9 cm^2^. The images for two representative cases are shown in Figure 3. Patients with stenosis more severe than this cutoff value and those with intervertebral instability were considered more likely to need additional surgery after TSCP.

Three other limitations to this study that should be noted. First, this study evaluated efficacy by focusing on whether the patients underwent open revision surgery after TSCP and did not follow improvement of clinical features such as VAS over the long term as other studies have done. Second, this was a retrospective study, so it may not have included all patients with severe stenosis and would not include patients who preferred open surgery rather than TSCP from the outset. Therefore, the exact extent to which TSCP can avoid open revision surgery could not be determined. Also, we evaluated whether reoperation was avoided during a short follow-up period. Third, because the surgeries were performed by a variety of surgeons during the study period, we cannot rule out the possibility that the results were affected by unrecognized effects of individual differences between surgeons, such as patient selection bias or surgical indications. Therefore, there may have been cases in which TSCP was performed without adequate conservative treatment such as epidural block.

## Conclusion

In conclusion, 69.6% of patients who underwent TSCP during our study period were able to avoid open revision surgery. We did not identify any background factors, such as surgical history, age or BMI, that could have led to open revision surgery. However, the presence of intervertebral instability and a narrow dural sac area were found to be preoperative imaging factors leading to additional surgery. Although TSCP is a minimally invasive procedure and may avoid open revision surgery regardless of previous surgical history, open surgery should be the first choice for patients with intervertebral instability or severe spinal canal stenosis.

## Data Availability

The raw data supporting the conclusions of this article will be made available by the authors, without undue reservation.

## References

[1] Ishii K Watanabe G Tomita T Nikaido T Hikata T Shinohara A Minimally invasive spinal treatment (MIST)-A new concept in the treatment of spinal diseases: a narrative review. Medicina. (2022) 58(8):1123. 10.3390/medicina58081123 36013590 PMC9413482

[2] Momin AA Steinmetz MP. Evolution of minimally invasive lumbar spine surgery. World Neurosurg. (2020) 140:622–6. 10.1016/j.wneu.2020.05.071 32434014

[3] Lykissas MG Giannoulis D. Minimally invasive spine surgery for degenerative spine disease and deformity correction: a literature review. Ann Transl Med. (2018) 6(6):99. 10.21037/atm.2018.03.18 29707548 PMC5900066

[4] Huang TJ Kim KT Nakamura H Yeung AT Zeng J. The state of the art in minimally invasive spine surgery. Biomed Res Int. (2017) 2017:6194016. 10.1155/2017/6194016 28337454 PMC5350391

[5] Hopkins B Mazmudar A Kesavabhotla K Patel AA. Economic value in minimally invasive spine surgery. Curr Rev Musculoskelet Med. (2019) 12(3):300–4. 10.1007/s12178-019-09560-8 31236835 PMC6684673

[6] Allen RT Garfin SR. The economics of minimally invasive spine surgery: the value perspective. Spine. (2010) 35(26):S375–82. 10.1097/BRS.0b013e31820238d9 21160403

[7] Moon BJ Yi S Ha Y Kim KN Yoon DH Shin DA. Clinical efficacy and safety of trans-sacral epiduroscopic Laser decompression compared to percutaneous epidural neuroplasty. Pain Res Manage Pain Res Manag. (2019) 2019:2893460. 10.1155/2019/2893460 PMC6348914 30755783

[8] Funao H Yokosuka K Ukai J Nakanishi K Paku M Tomita T Efficacy of minimally invasive trans-sacral canal plasty between patients with and without failed back surgery syndrome. Medicina. (2022) 58(2):251. 10.3390/medicina58020251 35208574 PMC8879517

[9] Urits I Schwartz RH Brinkman J Foster L Miro P Berger AA An evidence based review of epidurolysis for the management of epidural adhesions. Psychopharmacol Bull. (2020) 50(4 Suppl 1):74–90 33633419 10.64719/pb.4383 PMC7901122

[10] Manchikanti L Knezevic NN Sanapati MR Boswell MV Kaye AD Hirsch JA. Effectiveness of percutaneous adhesiolysis in managing chronic central lumbar spinal stenosis: a systematic review and meta-analysis. Pain Physician. (2019) 22(6):E523–e550. 10.36076/ppj/2019.22.E523 31775400

[11] Chun-jing H Hao-xiong N Jia-xiang N. The application of percutaneous lysis of epidural adhesions in patients with failed back surgery syndrome. Acta Cir Bras. (2012) 27(4):357–62. 10.1590/s0102-86502012000400013 22534813

[12] Manchikanti L Cash KA McManus CD Pampati V. Assessment of effectiveness of percutaneous adhesiolysis in managing chronic low back pain secondary to lumbar central spinal canal stenosis. Int J Med Sci. (2013) 10(1):50–9. 10.7150/ijms.5303 23289005 PMC3534877

[13] Arimura D Shinohara A Katsumi S Obata S Ikegami T Saito M. Transsacral canal plasty for decompression of lumbar spinal stenosis in a patient with epidural lipomatosis: a case report. JBJS Case Connect. (2022) 12(4):e22.00494. 10.2106/jbjs.Cc.22.00494 36656263

[14] Kuligowski T. Prevalence of lumbar segmental instability in young individuals with the different types of lumbar disc herniation-preliminary report. Int J Environ Res Public Health. (2022) 19(15):9378. 10.3390/ijerph19159378 35954735 PMC9368739

[15] Menendez ME Ring D Harris MB Cha TD. Predicting in-hospital mortality in elderly patients with cervical spine fractures: a comparison of the charlson and elixhauser comorbidity measures. Spine. (2015) 40(11):809–15. 10.1097/brs.0000000000000892 25785957

[16] van Walraven C Austin PC Jennings A Quan H Forster AJ. A modification of the Elixhauser comorbidity measures into a point system for hospital death using administrative data. Med Care. (2009) 47(6):626–33. 10.1097/MLR.0b013e31819432e5 19433995

[17] Elixhauser A Steiner C Harris DR Coffey RM. Comorbidity measures for use with administrative data. Med Care. (1998) 36(1):8–27. 10.1097/00005650-199801000-00004 9431328

[18] Helm S 2nd Racz GB Gerdesmeyer L Justiz R Hayek SM Kaplan ED Percutaneous and endoscopic adhesiolysis in managing low back and lower extremity pain: a systematic review and meta-analysis. Pain Physician. (2016) 19(2):E245–82. 10.36076/ppj/2016.19.E245 26815254

[19] Heavner JE Racz GB Raj P. Percutaneous epidural neuroplasty: prospective evaluation of 0.9% NaCl versus 10% NaCl with or without hyaluronidase. Reg Anesth Pain Med. (1999) 24(3):202–7. 10.1016/s1098-7339(99)90128-1 10338168

[20] Racz GB Heavner JE Trescot A. Percutaneous lysis of epidural adhesions–evidence for safety and efficacy. Pain Pract. (2008) 8(4):277–86. 10.1111/j.1533-2500.2008.00203.x 18503627

[21] Racz GB Heavner JE. Epidural phenol neurolysis using daily needle placements. Anesth Analg. (1986) 65(7):822–3. 10.1213/00000539-198607000-00025 2424344

[22] Ross JS Robertson JT Frederickson RCA Petrie JL Obuchowski N Modic MT Association between peridural scar and recurrent radicular pain after lumbar discectomy: magnetic resonance evaluation. Neurosurgery. (1996) 38(4):855–63. 10.1227/00006123-199604000-00053 8692415

[23] Rabinovitch DL Peliowski A Furlan AD. Influence of lumbar epidural injection volume on pain relief for radicular leg pain and/or low back pain. Spine J. (2009) 9(6):509–17. 10.1016/j.spinee.2009.03.003 19398387

[24] Manchikanti L Knezevic NN Knezevic E Pasupuleti R Kaye AD Sanapati MR Efficacy of percutaneous adhesiolysis in managing low back and lower extremity pain: a systematic review and meta-analysis of randomized controlled trials. Pain Ther. (2023) 12(4):1–35. 10.1007/s40122-023-00508-y 37227685 PMC10209570

[25] Cho PG Ji GY Yoon YS Shin DA. Clinical effectiveness of percutaneous epidural neuroplasty according to the type of single-level lumbar disc herniation: a 12-month follow-up study. J Korean Neurosurg Soc. (2019) 62(6):681. 10.3340/jkns.2019.0070 31591998 PMC6835144

[26] Bellini M Barbieri M. A comparison of non-endoscopic and endoscopic adhesiolysis of epidural fibrosis. Anaesthesiol Intensive Ther. (2016) 48(4):266–71. 10.5603/AIT.a2016.0035 27595746

[27] Lee F Jamison DE Hurley RW Cohen SP. Epidural lysis of adhesions. Korean J Pain. (2014) 27(1):3–15. 10.3344/kjp.2014.27.1.3 24478895 PMC3903797

[28] Yokosuka K Sato K Yamada K Yoshida T Shimazaki T Morito S Computed tomographic epidurography in patients with low back pain and leg pain: a single-center observational study. Diagnostics. (2022) 12(5):1267. 10.3390/diagnostics12051267 35626422 PMC9141985

[29] Achttien RJ Powell A Zoulas K Staal JB Rushton A. Prognostic factors for outcome following lumbar spine fusion surgery: a systematic review and narrative synthesis. Eur Spine J. (2022) 31(3):623. 10.1007/s00586-021-07018-5 34705106

[30] Choi E Nahm FS Lee PB. Evaluation of prognostic predictors of percutaneous adhesiolysis using a Racz catheter for post lumbar surgery syndrome or spinal stenosis. Pain Physician. (2013) 16(5):E531–6. 24077203

[31] Lim YS Mun JU Seo MS Sang BH Bang YS Kang KN Dural sac area is a more sensitive parameter for evaluating lumbar spinal stenosis than spinal canal area: a retrospective study. Medicine. (2017) 96(49):e9087. 10.1097/md.0000000000009087 29245329 PMC5728944

[32] Sigmundsson FG Kang XP Jönsson B Strömqvist B. Prognostic factors in lumbar spinal stenosis surgery. Acta Orthop. (2012) 83(5):536–42. 10.3109/17453674.2012.733915 23083437 PMC3488183

